# Enhancing Treatment Decisions for Advanced Non-Small Cell Lung Cancer with Epidermal Growth Factor Receptor Mutations: A Reinforcement Learning Approach †

**DOI:** 10.3390/cancers17020233

**Published:** 2025-01-13

**Authors:** Hakan Şat Bozcuk, Leyla Sert, Muhammet Ali Kaplan, Ali Murat Tatlı, Mustafa Karaca, Harun Muğlu, Ahmet Bilici, Bilge Şah Kılıçtaş, Mehmet Artaç, Pınar Erel, Perran Fulden Yumuk, Burak Bilgin, Mehmet Ali Nahit Şendur, Saadettin Kılıçkap, Hakan Taban, Sevinç Ballı, Ahmet Demirkazık, Fatma Akdağ, İlhan Hacıbekiroğlu, Halil Göksel Güzel, Murat Koçer, Pınar Gürsoy, Bahadır Köylü, Fatih Selçukbiricik, Gökhan Karakaya, Mustafa Serkan Alemdar

**Affiliations:** 1 Independent Researcher, Antalya 07100, Turkey; 2 Private Practice, Burhanettin Onat Caddesi, 1419. Sokak, No:59, Ocean City, C-Blok, Kat:3, Daire:5, Muratpaşa, Antalya 07100, Turkey; 3 Department of Medical Oncology, Dicle University, Diyarbakır 21280, Turkey; leylasert21@hotmail.com (L.S.); drmalikaplan@hotmail.com (M.A.K.); 4 Department of Medical Oncology, Akdeniz University, Antalya 07058, Turkey; alimurattat@hotmail.com (A.M.T.); mustafakaraca@akdeniz.edu.tr (M.K.); 5 Department of Medical Oncology, Faculty of Medicine, İstanbul Medipol University, İstanbul 34810, Turkey; hm1635@hotmail.com (H.M.); ahmetknower@yahoo.com (A.B.); 6 Department of Medical Oncology, Necmettin Erbakan University, Konya 42090, Turkey; bilgesahkilictas@gmail.com (B.Ş.K.); mehmetartac@yahoo.com (M.A.); 7 Department of Medical Oncology, Marmara University, İstanbul 34722, Turkey; pinar.erel@marmara.edu.tr (P.E.); fyumuk@ku.edu.tr (P.F.Y.); 8 Division of Medical Oncology, School of Medicine, Koç University, İstanbul 34450, Turkey; bahadirkoylu@hotmail.com (B.K.); fsbiricik@yahoo.com (F.S.); 9 Faculty of Medicine, Ankara Yıldırım Beyazıt University, Ankara 06010, Turkey; drbbilgin@hotmail.com (B.B.); masendur@yahoo.com.tr (M.A.N.Ş.); 10 Department of Medical Oncology, Faculty of Medicine, İstinye University, İstanbul 34010, Turkey; skilickap@yahoo.com (S.K.); msalemdar@gmail.com (M.S.A.); 11 Department of Medical Oncology, Medical Park Keçiören Hospital, Ankara 06120, Turkey; hakantaban@yandex.com; 12 Department of Medical Oncology, Ankara University, Ankara 06100, Turkey; svncbll@gmail.com (S.B.); ademirka@gmail.com (A.D.); 13 Department of Medical Oncology, Sakarya University, Sakarya 54050, Turkey; fatmaakdag_87@hotmail.com (F.A.); ihacibekiroglu@sakarya.edu.tr (İ.H.); 14 Antalya Education and Research Hospital, Antalya 07100, Turkey; hgguzell@gmail.com (H.G.G.); muratkocer71@hotmail.com (M.K.); 15 Department of Medical Oncology, Ege University, İzmir 35040, Turkey; pinargursoy77@gmail.com; 16 Department of Medical Oncology, ASV Yaşam Hospital, Antalya 07300, Turkey; g.karakaya87@gmail.com

**Keywords:** non-small cell lung cancer, epidermal growth factor receptor, mutation, tyrosine kinase inhibitors, deep learning, machine learning, artificial intelligence

## Abstract

Background: Although higher-generation TKIs are associated with improved progression-free survival in advanced NSCLC patients with EGFR mutations, the optimal selection of TKI treatment remains uncertain. To address this gap, we developed a web application powered by a reinforcement learning (RL) algorithm to assist in guiding initial TKI treatment decisions. Methods: Clinical and mutational data from advanced NSCLC patients were retrospectively collected from 14 medical centers. Only patients with complete data and sufficient follow-up were included. Multiple supervised machine learning models were tested, with the Extra Trees Classifier (ETC) identified as the most effective for predicting progression-free survival. Feature importance scores were calculated by the ETC, and features were then integrated into a Deep Q-Network (DQN) RL algorithm. The RL model was designed to select optimal TKI generation and a treatment line for each patient and was embedded into an open-source web application for experimental clinical use. Results: In total, 318 cases of EGFR-mutant advanced NSCLC were analyzed, with a median patient age of 63. A total of 52.2% of patients were female, and 83.3% had ECOG scores of 0 or 1. The top three most influential features identified were neutrophil-to-lymphocyte ratio (log-transformed), age (log-transformed), and the treatment line of TKI administration, as tested by the ETC algorithm, with an area under curve (AUC) value of 0.73, whereas the DQN RL algorithm achieved a higher AUC value of 0.80, assigning distinct Q-values across four TKI treatment categories. This supports the decision-making process in the web-based ‘EGFR Mutant NSCLC Treatment Advisory System’, where clinicians can input patient-specific data to receive tailored recommendations. Conclusions: The RL-based web application shows promise in assisting TKI treatment selection for EGFR-mutant advanced NSCLC patients, underscoring the potential for reinforcement learning to enhance decision-making in oncology care.

## Simple Summary

Molecular targeted therapies have revolutionized the treatment of numerous cancers, including non-small cell lung cancer (NSCLC). Despite these advances, selecting the appropriate generation of tyrosine kinase inhibitors (TKIs) and determining the best treatment line remain complex challenges due to the wide array of available options. In this study, for the first time, we introduce the application of reinforcement learning—a state-of-the-art machine learning technique—to optimize systemic treatment strategies for patients with epidermal growth factor receptor (EGFR)-mutant advanced NSCLC, aiming to maximize progression-free survival. The core model was derived from a retrospective group of EGFR-mutant NSCLC cases treated at 14 medical centers. Building on this research, we created an experimental web application, freely available as an open-access tool, which serves as a novel treatment recommendation system. This platform highlights critical predictive features while demonstrating the potential of artificial intelligence to advance thoracic oncology care.

## 1. Introduction

Molecular targeted therapies have profoundly transformed the treatment landscape for various cancers, including non-small cell lung cancer (NSCLC) [1]. Following evidence demonstrating the superiority of tyrosine kinase inhibitors (TKIs) over chemotherapy for advanced NSCLC with epidermal growth factor receptor (EGFR) mutations, the treatment landscape has rapidly advanced with the introduction of newer EGFR TKIs, such as Osimertinib [2,3,4]. Importantly, studies have shown that EGFR TKIs extend both progression-free survival (PFS) and overall survival (OAS) compared to comparator arms in trials involving EGFR-mutant advanced NSCLC patients [5].

However, choosing the optimal TKI generation and treatment line remains challenging, especially with multiple options available, making it difficult to prescribe the most effective anti-EGFR TKI treatment for this patient group. Additionally, data indicate that the benefits of EGFR TKI treatment vary based on patient and disease characteristics and the specific type of EGFR mutation [6]. Furthermore, access to EGFR testing may be limited or delayed in certain settings, impacting the timing and choice of EGFR TKI treatment [7]. Consequently, in real-world practice, prescribing the ideal EGFR TKI therapy for patients with EGFR-mutant advanced NSCLC is a complex and highly individualized task.

Traditional oncology treatments have largely relied on clinical guidelines derived from population-level data. However, recent advancements in machine learning (including the use of decision trees such as Extra Trees Classifier (ETC), boosting algorithms, etc.), particularly reinforcement learning (RL), provide opportunities for more personalized treatment recommendations by analyzing individual patient characteristics and clinical histories. Falling under the broader scope of AI, RL is gaining traction in oncology for optimizing treatment decisions and personalizing patient care. Unlike traditional models, RL learns dynamically through iterative feedback, using reward-based mechanisms to determine optimal strategies for complex, sequential decision-making, such as selecting the most effective treatment pathway for cancer patients. RL models, including Deep Q-Networks (DQN) and Policy Gradient methods, have demonstrated potential in guiding adaptive therapies by balancing treatment efficacy with side-effect management [8,9]. By simulating possible treatment outcomes, RL can assist oncologists in tailoring therapies more closely to each patient’s needs, ultimately improving survival rates and quality of life [10]. This study introduces a RL-based approach to guide TKI treatment selection for advanced EGFR-mutant NSCLC, incorporating patient, disease, and treatment features, along with the neutrophil-to-lymphocyte ratio (NLR)—a biomarker reflecting the balance between inflammatory and immune responses. As a result, the model has been implemented as a web application to provide real-time clinical decision-making support.

## 2. Materials and Methods

### 2.1. Data Collection and Engineering

Clinical and mutational data for advanced NSCLC patients with EGFR mutations were retrospectively collected by investigators from both online and offline databases and then combined from 14 participating medical centers. Only patients with metastatic, advanced disease who carried EGFR mutations, with complete clinical and mutational records were included, with follow-up criteria requiring at least one year for those progression-free or shorter if progression or death occurred within the first year following TKI initiation. Also, patients with a very short follow-up period, i.e., a follow-up period of less than one month of follow-up after initial TKI administration, were not included in this study. This curated dataset was then used for model construction and evaluation, ensuring that the analysis was based on comprehensive and clinically relevant information.

The collected data contained information on age, gender, Eastern Cooperative Oncology Group (ECOG) performance status, the type of EGFR mutation, the treatment line of first TKI administration, the name and generation of first TKI used, the number of metastatic sites, the presence of bone or liver metastases, the presence of brain metastases, the presence of significant comorbidity (coronary heart disease, chronic obstructive lung disease, renal or liver failure, Diabetes Mellitus, etc.), smoking status, and pre TKI treatment neutrophil and lymphocyte counts. The study participants were selected retrospectively without any limitations regarding the recruitment timeframe. Data engineering was conducted using these features to create new variables, two of which were retained for subsequent modeling. These included a logarithmic transformation of the neutrophil-to-lymphocyte ratio (NLR) and an “action” variable composed of four categories, resulting from the interaction between the treatment line and the type of first TKI administered. The action variable categories were as follows: 0; first-line, first-generation TKI, 1; first-line, second- or higher-generation TKI, 2; second- or later-line, first-generation TKI, 3; and second- or later-line, second- or higher-generation TKI. The NLR was assessed within the month immediately preceding the initiation of the first tyrosine kinase inhibitor (TKI) treatment to ensure an unbiased and timely measurement.

A considerable proportion of cases did not document death as an endpoint, and missing data led to the exclusion of 34% of the population. Consequently, overall survival was not chosen as an endpoint for this study. Instead, progression-free survival (PFS) was selected as the primary endpoint of interest. For the outcome of PFS, any progression or death event was considered significant. To support subsequent phases of machine learning modeling, a dichotomous variable (progression category) was created, indicating whether or not patients achieved a progression-free survival duration of one year or more.

The line of initial tyrosine kinase inhibitor (TKI) administration in our cohort was highly variable. To assess the impact of each additional TKI line on outcomes without dividing cases into multiple categories, we treated this variable as quantitative in our machine learning models, emphasizing feature importance and overall explainability. However, since the line of initial TKI administration emerged as one of the top three most influential factors in the ETC model and holds significant clinical relevance in the treatment pathway, we handled it differently in our reinforcement learning (RL) algorithm. For the RL model, which aims to provide practical treatment recommendations to enhance the likelihood of being progression-free beyond one year, we dichotomized the line of initial TKI administration to establish a clinically meaningful scenario.

This study was planned and conducted as a project of the Turkish Oncology Group (TOG) Lung Cancer Subgroup, with a preliminary version presented as a poster at the 2024 European Society for Medical Oncology (ESMO) meeting [11]. Our current work expands on the previous study by including additional cases and employing a different research question, objective, and methodology. To enhance readability, we utilized ChatGPT-4 for manuscript drafting; however, as authors, we reviewed and refined the content as needed and take full responsibility for the publication’s content.

### 2.2. Extra Trees Classifier and Feature Importance Analysis

To calculate individual feature importance values, 15 machine learning models were tested using the PyCaret library, which enables the application of cutting-edge machine learning algorithms to tabular data [12]. Among these models, ETC demonstrated the highest performance. Therefore, ETC was selected for feature importance analysis and to generate the feature importance plot. The binary outcome variable used was the progression category, as defined previously. Due to the imbalanced nature of the outcome variable, the imblearn library was used to apply the Synthetic Minority Over-sampling Technique (SMOTE) [13,14], a technique in machine learning that addresses class imbalance by creating synthetic samples for the minority class, thus enhancing model reliability on imbalanced datasets. The coding and analysis were conducted in Google Colab [15].

### 2.3. Reinforcement Learning Model Development

The Gymnasium library was employed for the development of the reinforcement learning (RL) model. Gymnasium is an open-source toolkit designed for creating and comparing RL algorithms, offering a standardized interface for a wide range of environments where RL agents can be trained and tested [16]. This library includes a variety of benchmarks commonly used in RL research, supports multiple environments, and integrates with other tools, making it an adaptable platform for RL model experimentation and enhancing reproducibility across different algorithms and tasks. In our study, a simulated, programmed agent interacted with an environment to achieve a specific goal: to determine the optimal generation and treatment line of TKI for a given case. The objective was to identify the best “action” for each patient, represented by an action variable that defines the model’s action space (four possible actions: Actions 0, 1, 2, and 3). Q values were generated for each action, based on clinical outcomes, guiding the model’s decision-making process. The action variable was previously detailed in the Data Collection and Engineering section.

We also employed the Boltzmann exploration technique in our reinforcement learning model to optimize action selection [17]. This technique is especially valuable in reinforcement learning as it balances exploration (testing new actions) with exploitation (selecting actions that have previously shown high rewards). Boltzmann exploration relies on a probabilistic approach, where actions are chosen based on their Q-values, and the probability of selecting each action is weighted by its Q-value after applying a “temperature” parameter, tau. We manually tested different tau values to identify the optimal balance for our RL model. Additionally, we branched the RL algorithm according to the line of first TKI treatment, as this feature was among the top three most influential factors in the ETC model and is clinically significant in the treatment pathway. Thus, we designed separate scenarios for first-line TKI use and for second-line or later TKI use, prioritizing cases where second-generation or higher TKIs were administered. This was achieved by adjusting and testing reward points within the action space, as using higher-generation TKIs is linked to improved progression-free survival (PFS) outcomes [18].

We utilized a Deep Q-Network (DQN) as our reinforcement learning approach, employing Multi-Layer Perceptron—a type of neural network composed of interconnected layers—as its decision-making framework [19,20]. The model was trained over 50,000 timesteps, during which it interacted with the treatment environment by assessing each patient’s current condition, selecting an appropriate EGFR TKI therapy, and receiving feedback in the form of rewards to enhance its treatment choices. Throughout the training process, the DQN applied Q-learning to refine its strategy by estimating the expected benefits (Q-values) of each treatment option for a given patient profile and adjusting its neural network to improve these estimates. Over time, the model learned to select therapies that maximized overall patient outcomes, specifically aiming to improve PFS. Additionally, we generated partial dependence plots to illustrate how different treatment options interact with patient characteristics, analyzed the frequency of each treatment choice, and calculated summary Q-values for each EGFR TKI option.

### 2.4. EGFR Mutant NSCLC Treatment Advisory System and Web Application Deployment

The reinforcement learning model, named the EGFR Mutant NSCLC Treatment Advisory System, was embedded into a web application using Streamlit, allowing oncologists to input patient data and receive real-time treatment recommendations [21]. The web application was developed separately using the Spyder integrated development environment within the Anaconda framework [22,23]. The interface offers a user-friendly layout where clinicians can enter patient demographics, mutational status, and other relevant clinical details. For ease of access and scalability, the application was hosted on a cloud platform via Streamlit.

## 3. Results

### 3.1. General Features and Feature Importance Analysis

Data were collected from 14 institutions, comprising 481 cases of advanced NSCLC with EGFR mutations. Patients in our study were recruited over a twelve-year period, spanning from 2013 to 2024. However, 163 cases (34%) were excluded due to inadequate follow-up or missing data. As a result, a total of 318 EGFR mutant advanced NSCLC cases with complete data and sufficient follow-up were recruited. The median age was 63, 52.2% were female, and 83.3% had an ECOG performance score of 0 or 1. The most common treatment scenario involved the use of a first-generation TKI as the line of systemic treatment. Erlotinib was the most prescribed EGFR TKI, used in 73.3% of cases. The median follow-up time was 16.5 months, with an Interquartile Range (IQR) of 22.2 months. See Table 1 for patient, disease, and treatment details. Also, note that the Supplementary File, Table S1, includes the database for cases recruited in this study.

**Figure 1:**
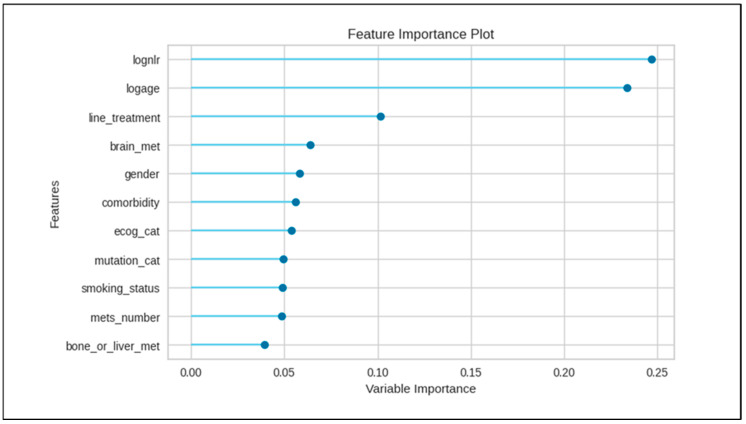
Feature importance plot. Variable importance figures for features in the study. Lognlr, logarithmic transformation of neutrophil-to-lymphocyte ratio; logage, logarithmic transformation of age; line_treatment, line of initial TKI usage; brain_met, presence or absence of brain metastases; ecog_cat, ECOG performance status; mutation_cat, Exon 19 mutation or other mutations; smoking_status, never or ever smoker; mets_number, number of metastases up to three or more; bone_or_liver_met, presence or absence of bone and/or liver metastases.

The initial distribution of the progression category for our cases on TKI treatment consisted of 196 cases with progression and 122 cases without progression. After applying the Synthetic Minority Over-sampling Technique (SMOTE), the class distribution was balanced, resulting in 196 cases with progression and 196 cases without progression. This adjustment led to the generation of 74 synthetic cases for the progression-absent category, which accounted for 38% of the progression-absent group (74 out of 196 cases). No synthetic samples were created for the progression-present category. Among the 15 machine learning models evaluated, ETC achieved the highest performance, with an area under the curve (AUC) of 0.73 and an accuracy score of 0.66. AUC values across the models ranged from 0.51 to 0.73. Detailed performance metrics and rankings for all the models are presented in Table 2. The ETC model was used for the feature importance analysis, identifying the top three important features as the logarithmic transformation of NLR, the logarithmic transformation of age, and the line of first TKI use, with importance scores ranging from 0.10 to 0.25. The feature importance plot is shown in Figure 1. Notably, the NLR values did not significantly differ based on progression status; an independent samples t-test indicated no statistical difference between early and late progression cases (t = −1.02, df = 316, *p* = 0.308).

For modeling and training, we utilized 90% of the SMOTE-enhanced dataset, comprising 353 out of 392 cases. As mentioned above, our TKI treatment cohort initially included 196 cases with progression and 122 cases without progression. To balance the groups, we applied SMOTE, generating 74 synthetic cases and increasing the number of non-progression cases to 196. This resulted in a total of 392 cases (196 with progression and 196 without progression). The selected 353 cases were then used for modeling and training, ensuring a balanced and representative dataset.

### 3.2. Reinforcement Learning Model and EGFR Mutant NSCLC Treatment Advisory System

Our DQN model was implemented using the stable-baselines3 library with the MlpPolicy, which comprises a multi-layer perceptron tailored for our specific application. The architecture consists of an input layer matching the number of standardized patient features, followed by two hidden layers, each containing 64 neurons with Rectified Linear Unit (ReLU) activation functions, and an output layer with four neurons corresponding to the four possible tyrosine kinase inhibitor (TKI) treatment actions. This structure was selected to ensure sufficient capacity for learning complex patterns in the data while maintaining computational efficiency and preventing overfitting. For hyperparameter optimization, we employed a systematic tuning strategy to identify the most effective settings for our DQN model. Key hyperparameters, including the learning rate, discount factor (γ), batch size, and exploration temperature (τ), were initially set based on values commonly reported in the literature and subsequently fine-tuned through iterative experimentation.

To determine the most suitable model for treatment recommendations, we compared the efficacy of the ETC and RL models for developing the NSCLC treatment advisory system. The RL model achieved a higher AUC (0.80 vs. 0.73 for ETC), indicating superior global ranking of treatment recommendations, which is the system’s primary goal. In contrast, ETC showed a better F1 score (0.65 vs. 0.60), reflecting improved precision-recall balance. Considering the RL model’s nearly 10% higher AUC, we selected it as the foundation for the EGFR Mutant NSCLC Treatment Advisory System.

**Figure 2:**
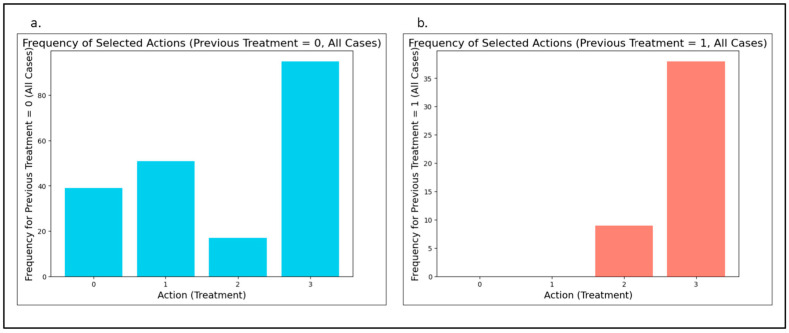
Action frequencies with respect to the line of first TKI treatment. Action frequency figures at first and subsequent lines of first TKI treatment (in chemotherapy naïve and refractory patients). Action definitions: 0; first-line, first-generation TKI, 1; first-line, second- or higher-generation TKI, 2; second- or later-line, first-generation TKI, 3; second- or later-line, second- or higher-generation TKI. (**a**). Action frequencies at first line (chemotherapy naïve patients). (**b**). Action frequencies at second or subsequent line (chemotherapy refractory patients).

For patients receiving TKI as a first-line treatment (chemotherapy naïve), the RL model predominantly recommended a second- or higher-generation TKI. Similarly, when TKI was prescribed as a second- or later-line treatment (for chemotherapy refractory patients), the model also favored second- or higher-generation TKIs. Figure 2 displays the frequency of recommended actions in relation to the line of first TKI use. When the line of TKI administration was not considered, the model’s Q values most frequently supported Action 3—administering a second- or later-line, second- or higher-generation TKI —over the other three actions (Actions 0, 1, and 2), with a mean Q value of 14.7. Table 3 provides detailed Q values for each recommended action.

**Figure 3:**
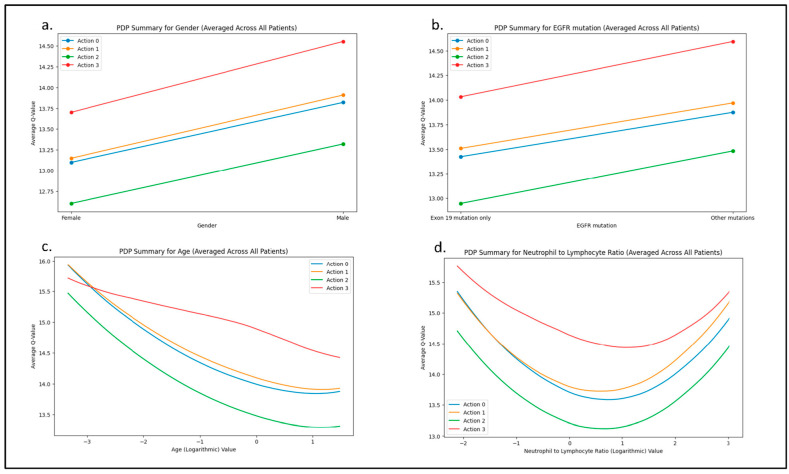
Partial dependence plots. Visualizing action–feature interactions, plotting action frequencies, and calculating summary Q-values for each action within the RL model. (**a**). Gender and actions. (**b**). Type of EGFR mutation and actions. (**c**). Age (logarithmic transformation) and actions. (**d**). NLR (logarithmic transformation) and actions.

Partial dependence plots examining the distribution of actions relative to other features revealed that gender did not influence the type of recommendation, typically Action 3 (second- or later-line, second- or higher-generation TKI). However, male gender was associated with higher Q values. Similarly, the type of EGFR mutation did not alter the recommendation between Action 3 or 1, though Q values tended to be higher for mutations other than Exon 19. Age also influenced the recommendations (Action 1 or 0 were more strongly recommended for younger patients, whereas Action 3 was favored for older patients). Regarding the NLR, no interaction was observed with the primary recommendation, Action 3. However, the second-best recommendation showed a marginal preference for Action 0 when the NLR was lower, whereas Action 1 was favored when the NLR was higher. Selected partial dependence plots are shown in Figure 3a–d.

**Figure 4:**
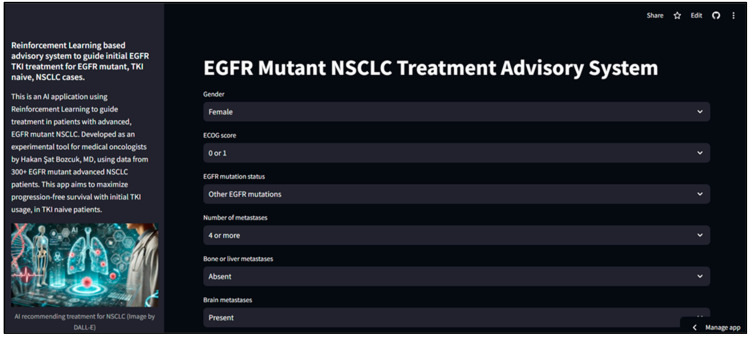
Web application interface. The web application, named “EGFR Mutant NSCLC Treatment Advisory System” and its interface, accessible at https://egfr-recommender.streamlit.app/, accessed on 6 January 2025.

The reinforcement learning (RL) algorithm was subsequently used to develop a web application called the “EGFR Mutant NSCLC Treatment Advisory System”, intended for experimental clinical use and accessible at “https://egfr-recommender.streamlit.app/, accessed on 6 January 2025”. This application utilizes the RL algorithm to generate treatment recommendations for new advanced NSCLC cases with EGFR mutations, with the goal of optimizing progression-free survival (PFS). Since the application is deployed on a free platform, there may be occasional pauses when bringing it online. The user interface of the application is shown in Figure 4.

## 4. Discussion

In this study, we demonstrated the feasibility of using reinforcement learning (RL) to guide treatment decisions for advanced EGFR-mutant NSCLC patients, with the objective of optimizing progression-free survival (PFS). We also successfully implemented this algorithm in a freely accessible web application, available for experimental use by medical oncologists. To our knowledge, this is one of the first reported applications of RL as a treatment recommendation system specifically for targeted cancer therapy. RL is already seeing broader use in oncology, with applications in radiology for breast cancer screening and liver lesion characterization, radiation oncology for prostate cancer IMRT treatment planning, adaptive therapy for NSCLC, and beam orientation in Cyberknife treatment planning [24]. Other emerging uses of RL in oncology include prostate biopsy planning, drug discovery, and simulated clinical trials [25,26]. Thus, our study contributes to the growing body of research supporting the potential for RL use in cancer screening, diagnosis, and treatment.

Various machine learning models, aside from RL, have been applied to guide targeted cancer therapies; however, these efforts have primarily focused on target identification, virtual screening for drug design, and identifying genes and proteins linked to treatment outcomes. Additionally, machine learning has been employed to analyze genomics, transcriptomics, proteomics, radiomics, digital pathology images, and other complex data, providing clinicians with a synthetic and comprehensive understanding of tumors rather than direct treatment recommendations, as in our study [26,27]. Building on this foundation, we integrated a RL algorithm into a web-based platform, presenting a practical, patient-focused tool that assists clinicians in selecting tailored treatments for advanced EGFR-mutant NSCLC. This tool identifies EGFR TKI options that are more likely to improve progression-free survival PFS by taking into account the patient’s unique clinical features, disease characteristics, and specific EGFR mutation profiles.

Recommendation systems are tools that suggest items or actions based on data reflecting user preferences, behaviors, or specific characteristics. Traditionally used in fields such as e-commerce or entertainment (e.g., product or movie recommendations), recommendation systems are gaining traction in healthcare. In this setting, they analyze large datasets on patient characteristics, treatments, and outcomes to make personalized recommendations, supporting clinical decision-making. Similarly, our study presents a recommendation system for patients with EGFR-mutant NSCLC, utilizing RL to recommend the optimal generation and line of first EGFR TKI treatment. By analyzing patient-specific data, including age, performance status, prior treatments, and other clinical attributes, the system aims to provide treatment recommendations tailored to maximize progression-free survival and improve overall treatment effectiveness, supporting oncologists in their daily clinical practice. Other treatment recommendation systems in medicine make use of various algorithms, generally other than RL. For example, Alian et al. used a personalized recommender based on forward chaining reasoning, in order to support diabetes management, and Zhang, et al. made use of the Gated Recurrent Unit (GRU) to produce a personalized interpretable deep representation of longitudinal electronic health records [28,29]. So, we have been able to make use of the underutilized RL approach for our treatment recommendation system with good effect. Our findings highlight the vast potential of RL algorithms to guide clinical practice in medicine in general, and in oncology specifically.

In our study, the RL model proved effective for making dynamic treatment decisions. It utilized Q-learning and Boltzmann exploration to simulate and optimize different treatment pathways, enabling personalized recommendations that adapt to each patient’s changing condition. Meanwhile, the ETC efficiently analyzed our dataset by addressing data imbalances with SMOTE and identifying key predictive factors. For our purposes, the ETC was particularly valuable in determining which features were most important for predicting treatment outcomes. In contrast, the RL model provided the added benefit of offering real-time, dynamic, and adaptable treatment suggestions, as demonstrated in our web-based application for managing EGFR-mutant advanced NSCLC. Together, these methods complemented each other. The ETC guided the selection of important features, whereas the RL model delivered customized and flexible treatment recommendations tailored to each patient’s unique clinical profile.

It is crucial to emphasize that although our RL model recommended higher-generation TKIs in most instances, consistent with current clinical guidelines, it identified specific subsets of patients within both chemotherapy naïve and refractory cohorts who are predicted to achieve better progression-free survival with first-generation TKIs compared to higher-generation TKIs. This finding was not observed in randomized clinical trials, representing a novel contribution of our research. Consequently, our RL model not only aligns with the outcomes of established randomized controlled trials, but also reveals variations influenced by the unique clinical and disease characteristics of individual patients. However, amid the increasing integration of AI in healthcare, our RL model and accompanying web application are designed to assist, not replace, medical practitioners by providing RL-based treatment recommendations. Ultimately, clinicians retain full authority over treatment decisions, ensuring that human expertise and individualized patient considerations—aligned with international guidelines and findings from randomized controlled trials—remain central to medical care.

The neutrophil-to-lymphocyte ratio (NLR) emerged as one of the top influential features in the ETC model in our study. Although few studies have explored NLR as a predictive factor for metastatic NSCLC patients treated with EGFR TKIs, those that have suggest that a high NLR is associated with a worse prognosis [30,31]. In our study, although NLR values did not significantly differ between early- and late-progressing groups, the ETC model ranked it as the third most important feature. It is essential to note that feature importance in machine learning is model-specific, meaning the score reflects the feature’s contribution within the structure of that particular model. High importance indicates that NLR significantly enhances prediction accuracy in the ETC model, though it does not establish causation. We consider NLR an important predictor for treatment selection in this setting, as demonstrated by our ETC model. However, it likely interacts with other predictive features in complex ways that remain to be fully understood in this patient population.

Our study has several limitations. Although a sample size of 318 cases without missing data is significant, larger datasets in the thousands would likely enhance model accuracy, as increased sample sizes are associated with improved precision in machine learning models [32]. However, although our sample size is relatively modest for machine learning and reinforcement learning applications, we consider this study to be a significant initial effort. It lays the groundwork for future research that will analyze thousands of cases using AI models with a comprehensive set of predictor features. Additionally, the retrospective design of our study introduces some risk of confounding and bias compared to prospective studies [33]. Another limitation is our use of progression-free survival (PFS) as the endpoint. Although PFS is appropriate, overall survival would offer a more comprehensive measure of treatment efficacy if patients were followed for a longer duration and a sufficient number of events occurred. Furthermore, only a small proportion of patients in our study received a third-generation EGFR TKI, with the majority treated using first- or second-generation TKIs. This treatment distribution makes it more challenging to generalize our findings across all generations of EGFR TKIs. Certainly, incorporating a greater number of cases involving the use of third-generation EGFR TKIs could have enhanced the accuracy of the AI models utilized in this study.

Lastly, this study does not report on TKI toxicity, which is an important factor in treatment selection, and should also be addressed in future studies. Future research with a larger, prospectively designed cohort, a longer follow-up period, overall survival as the primary endpoint, and a more balanced representation of patients treated with all EGFR TKI generations would likely yield more accurate models and more reliable treatment recommendations. Nonetheless, we believe this study provides a strong foundation for such future investigations. Also, we acknowledge that prospective validation is crucial to establish the tool’s effectiveness and reliability in a real-world clinical setting. Although our current study focuses on the development and retrospective evaluation of the tool, we recognize the need for future prospective studies. We plan to collaborate with clinical partners to conduct prospective validation studies, which will assess the tool’s performance, usability, and impact on clinical decision-making in real-time.

In summary, our study demonstrates the feasibility and potential of using RL as a treatment recommendation system for EGFR-mutant advanced NSCLC patients undergoing EGFR TKI therapy. By deploying this RL model through a web app, we introduce a practical tool for clinicians, supporting easy integration into clinical workflows. We believe this study highlights the broader applicability of RL in guiding diagnosis and treatment for NSCLC and potentially for other cancers. Specifically, our work presents a novel approach and tool to assist in the targeted treatment of EGFR-mutant advanced NSCLC.

## 5. Conclusions

Our study highlights the potential of RL as a decision-support system for recommending EGFR TKI therapies in patients with EGFR-mutant advanced NSCLC. Through the development of a user-friendly web application, we present an experimental tool with the potential to guide clinical workflows. This research highlights the wider potential of RL in enhancing therapeutic decision-making for advanced EGFR-mutant NSCLC and other cancers. Ultimately, our work introduces a novel approach to enable personalized treatment strategies for EGFR-mutant advanced NSCLC.

## Figures and Tables

**Table 1:** Patient and treatment details.

Features		n	%	Mean	Standard Deviation	Percentile 50	Minimum	Maximum
Total		318	100.0					
*Patient Features*								
Age				62.7	11.4	63	31	92
Gender								
	Male	152	47.8					
	Female	166	52.2					
ECOG performance status								
	0 or 1	265	83.3					
	2 to 4	53	16.7					
Smoking status								
	Currently not smoking	201	63.2					
	Currently smoking	117	36.8					
Mutation category								
	Exon 19 mutation only	204	64.2					
	Exon 21 and/or other mutations	114	35.8					
Number of metastases								
	1 to 3	148	46.5					
	4 or more	170	53.5					
Bone and/or lver metastasis								
	Absent	137	43.1					
	Present	181	56.9					
Brain metastasis								
	Absent	200	62.9					
	Present	118	37.1					
Neutrophil-to-Lymphocyte Ratio (NLR)				3.9	3.2	3.2	1	25
	Present	512	10.2					
	Absent	4500	89.8					
*TKI Usage Details*								
Line of initial TKI administration				1.3	0.5	1	1	5
Name of initial TKI								
	Erlotinib	233	73.3					
	Gefitinib	27	8.5					
	Afatinib	45	14.2					
	Osimertinib	10	3.1					
	Dacomatinib	3	0.9					
Initial TKI utilization in relation to line of administration								
	1st line, 1st generation TKI	202	63.5					
	1st line, 2nd or higher generation TKI	47	14.8					
	2nd or higher line, 1st generation TKI	15	4.7					
	2nd or higher line, 2nd or higher generation TKI	54	17.0					
Initial TKI utilization in relation to type of mutation								
	Exon 19 mutation only, 1st generation TKI	140	44.1					
	Other mutations, 1st generation TKI	64	20.1					
	Exon 19 mutation only, 2nd or higher generation TKI	77	24.2					
	Other mutations, 2nd or higher generation TKI	31	11.6					

Patient, treatment, and EGFR mutation details for the whole cohort of patients.

**Table 2:** Comparison of machine learning models.

Model	Accuracy	AUC	Recall	Prec.	F1	Kappa	MCC	TT (Sec)
Extra Trees Classifier *	0.66	0.73	0.65	0.67	0.65	0.32	0.33	0.06
Random Forest Classifier	0.65	0.72	0.66	0.65	0.65	0.30	0.30	0.08
Extreme Gradient Boosting	0.63	0.68	0.64	0.64	0.63	0.27	0.27	0.03
Light Gradient Boosting Machine	0.63	0.65	0.62	0.64	0.62	0.26	0.27	27.75
Gradient Boosting Classifier	0.63	0.67	0.67	0.63	0.64	0.26	0.26	0.04
Quadratic Discriminant Analysis	0.62	0.65	0.66	0.61	0.63	0.24	0.24	0.01
K Neighbors Classifier	0.61	0.64	0.57	0.63	0.59	0.22	0.22	0.02
Naive Bayes	0.60	0.62	0.62	0.61	0.61	0.20	0.20	0.02
Decision Tree Classifier	0.59	0.59	0.58	0.61	0.58	0.17	0.18	0.01
Ada Boost Classifier	0.58	0.63	0.61	0.60	0.60	0.16	0.16	0.03
Ridge Classifier	0.58	0.62	0.62	0.58	0.60	0.15	0.15	0.01
Logistic Regression	0.57	0.63	0.61	0.57	0.59	0.14	0.15	0.47
Linear Discriminant Analysis	0.56	0.61	0.61	0.57	0.58	0.13	0.13	0.01
SVM- Linear Kernel	0.53	0.57	0.71	0.58	0.58	0.07	0.10	0.01
Dummy Classifier	0.51	0.50	1.00	0.51	0.67	0.00	0.00	0.01

Performance metrics of the 15 machine learning models in this study to be employed for feature importance analysis. * Extra Trees Classifier with the highest performance metrics. The yellow color shows highest figures of efficacy for a selected efficacy measure.

**Table 3:** Q values with respect to actions.

	Action 0	Action 1	Action 2	Action 3
Mean	14.0	14.1	13.5	14.7
Standard deviation	1.0	1.0	1.0	1.2
Minimum	11.5	11.8	11.2	11.0
Maximum	16.6	16.8	16.1	17.0

Summary data for Q values with respect to the action recommended by the Reinforcement Learning model. Action definitions: 0; first-line, first-generation TKI, 1; first-line, second- or higher-generation TKI, 2; second- or later-line, first-generation TKI, 3; second- or later-line, second- or higher-generation TKI.

## Data Availability

The database and codebase are available upon reasonable request.

## Supplementary Materials

The following supporting information can be downloaded at: https://www.mdpi.com/article/10.3390/cancers17020233/s1, Table S1: Database for cases in the study.
